# Quantitative Measurement of Hexoses by Betaine Aldehyde Derivatisation

**DOI:** 10.3390/ijms27031446

**Published:** 2026-01-31

**Authors:** Paulina Kret-Bułat, Przemysław Mielczarek, Paweł Link-Lenczowski, Giuseppe Grasso, Piotr Suder, Anna Bodzon-Kulakowska

**Affiliations:** 1 Department of Analytical Chemistry and Biochemistry, Faculty of Materials Science and Ceramics, AGH University of Krakow, A. Mickiewicza 30, 30-059 Krakow, Poland; pkret@agh.edu.pl (P.K.-B.); piotr.suder@agh.edu.pl (P.S.); 2 Laboratory of Proteomics and Mass Spectrometry, Maj Institute of Pharmacology, Polish Academy of Sciences, Smetna 12, 31-343 Krakow, Poland; mielczar@if-pan.krakow.pl; 3 Department of Medical Physiology, Faculty of Health Sciences, Jagiellonian University Medical College, 31-126 Krakow, Poland; p.link-lenczowski@uj.edu.pl; 4 Center for the Development of Therapies for Civilization and Age-Related Diseases, Jagiellonian University Medical College, 31-066 Krakow, Poland; 5 Department of Chemical Sciences, University of Catania, Viale Andrea Doria 6, 95125 Catania, Italy; grassog@unict.it

**Keywords:** hexoses, MSI, betaine aldehyde, biomimetic calibration

## Abstract

Hexoses, particularly glucose, are one of the most essential molecules for sustaining life; therefore, reliable methods for their analysis are very important. In our study, we present a qualitative and quantitative approach for analysing hexoses using MALDI IMS (Matrix-Assisted Laser Desorption/Ionization Mass Spectrometry Imaging) with betaine aldehyde derivatisation and a CHCA (α-Cyano-4-hydroxycinnamic acid) matrix in positive ionisation mode. In this study, we demonstrated betaine aldehyde derivatisation of glucose from dried droplets and explored the analysis of hexoses in brain and liver tissue slices. We assessed whether our method could distinguish between mannose, galactose, glucose, and fructose and optimised the preparation of a biomimetic calibration curve using stable-isotope labelled glucose for hexose analysis. For this purpose, we investigated the number of betaine aldehyde layers required to obtain a proper calibration curve; examined whether changes in the spray nozzle position during CHCA matrix deposition could facilitate analysis and investigated how storage conditions influenced the calibration curve analysis. Finally, we optimised the technique for liver and brain analysis and assessed variations in hexose levels between brain, liver, kidney, and spinal cord tissues from control and morphine-addicted animals. We hope that our biomimetic approach to creating the calibration curve will be helpful for quantitative analysis and aid in developing various quantitative methods for assessing endogenous substances.

## 1. Introduction

Glucose, a simple sugar and the most biologically important hexose, is one of the most critical molecules for life. In particular, the mammalian brain depends on glucose as a primary source for ATP production, which is necessary for neuronal and non-neuronal cellular maintenance. Glucose metabolism fulfils important functions in the adult brain, including neuroenergetics, energy storage, biosynthesis, and oxidative defence. Additionally, neurotransmitter synthesis requires glucose as a precursor—a carbon source—due to the blood-brain barrier, and of course requires energy for this process [1]. Neurons are intolerant of an inadequate energy supply, and various pathologies are the consequence of disrupted energy metabolism. Several central nervous system disorders, such as epilepsy, spreading depression [2], axonal degeneration, Alzheimer’s disease [3], and Parkinson’s disease, are combined with disruption in energy metabolism (for an excellent review, see [4]). Of course, there are a lot of different biochemical processes that are based on energy metabolism, and different methods for glucose analysis are warmly welcomed.

Matrix-Assisted Laser Desorption/Ionisation Mass Spectrometry Imaging (MALDI MSI) is a robust technique that enables mapping the distribution of molecules in tissue slices [5]. Briefly, during this analysis, the tissue slice is placed on a special type of conductive ITO glass (Indium Tin Oxide glass), and the sample is covered with a matrix that facilitates the desorption and ionisation of the molecules upon exposure to a UV laser. At each spot, the mass spectrum is recorded, with specific *m*/*z* values and peak intensities associated with the amount of the molecule on the surface. Then, a map of the molecular distribution (an MSI image) is obtained by plotting the intensity of the peak characteristic for a given substance at each analysed point on the plane [6].

Obtaining spatial information is essential, especially in brain research, where different structures may be involved in different neurobiological processes. MALDI is usually used for the analysis of large molecules (e.g., lipids, proteins), but with a few requirements, it is possible to measure small molecules as well. There are specialised MALDI matrices that facilitate the measurement of specific molecules, but for some, a derivatisation process is required.

Betaine aldehyde reacts selectively with a hydroxyl group via nucleophilic addition to form a hemiacetal salt with a stable positive charge on a quaternary ammonium ion. That is why it could be used for the derivatisation of retinol, vitamin D2 and D3, L-ascorbic acid, glucose and cholesterol. This feature was shown for the first time by Wu et al. with a DESI (Desorption Electrospray Ionization) ion source [7]. We decided to test this approach with MALDI. Derivatisation technique allows, in this case, for ionization and analysis of glucose and other hexoses, and it enhances structural information, especially in the case of the brain.

Absolute quantification allows the determination of exact numerical concentrations of the analyte. To achieve this goal, a calibration curve with a known analyte concentration is required. In MSI experiments, three approaches are common: the first involves spotting a series of diluted standards on the glass slide without the tissue (off-tissue standard curve), the second requires spotting additional tissue sections with a series of diluted standards (on-tissue standard curve). The most demanding approach is the so-called biomimetic tissue model, in which tissue of the same type as the analysed sample is homogenised with varying amounts of the standard to generate an in-tissue standard series. This approach, although more demanding, is believed to best the analyte extraction from the tissue since it takes perfect account of the matrix effect and ion extraction efficiency (for an excellent review, see [8]). Since glucose and other hexoses are present in tissues, we need to use their stable-isotope labelled (SIL) versions. In animal tissues, hexoses are dominated by glucose; hence, only the glucose isotope was used in our study (additionally, see Section 3—Discussion).

Our proteomics meta-analysis of proteins involved in morphine addiction indicated that several enzymes with a role in energy metabolism are involved in this process [9]. Thus, in this study, we presented a fast and simple protocol for the derivatisation of glucose, tested whether this protocol could be useful for distinguishing other hexoses, and examined whether those molecules are involved in the brain and other tissues’ responses to morphine administration.

## 2. Results

### 2.1. Glucose Analysis from Dried Droplet and from the Tissue

**Figure 1:**
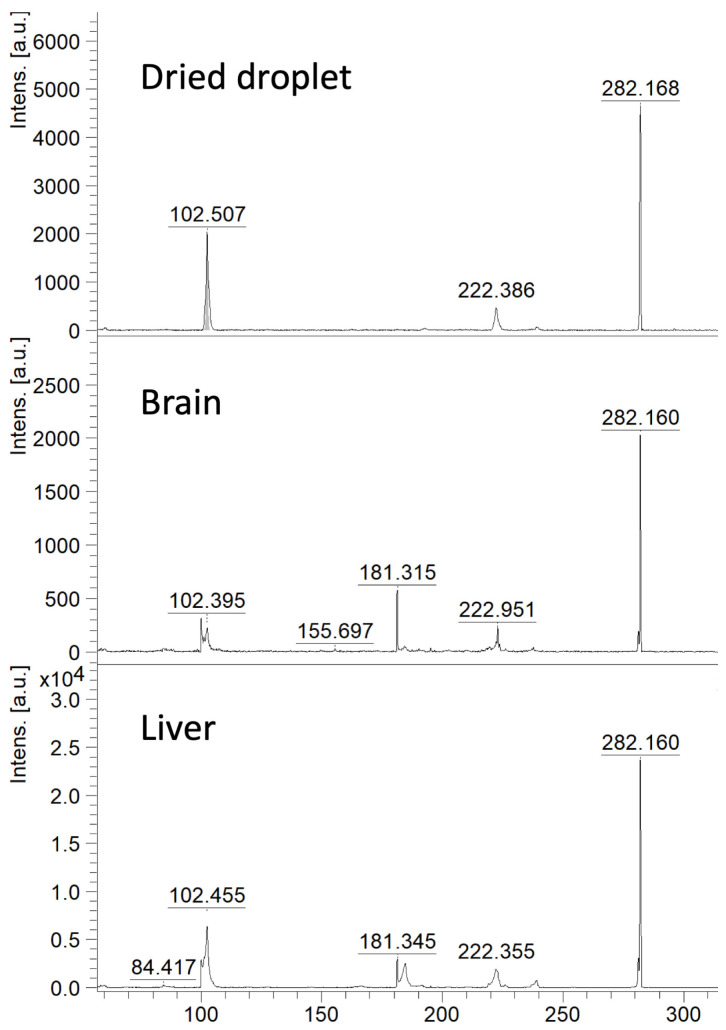
MALDI MS/MS spectra of derivatised glucose (*m*/*z* 282); detected using the dried-droplet approach and in brain and liver tissue sections following on-tissue chemical derivatisation with betaine aldehyde and CHCA matrix deposition.

Betaine aldehyde reacts selectively with the hydroxyl group. This reaction with glucose results in the formation of a derivatisation product at *m*/*z* = 282. We have performed dried-droplet and MSI analysis of brain tissue sections using betaine aldehyde derivatisation and a CHCA matrix (5 mg/mL ACN (acetonitrile): H_2_O, 2:1; 0.2% TFA (Trifluoroacetic Acid)) in the positive ion mode. Obtained MS/MS spectra were compared to respective MS/MS spectra from the tissue (see Figure 1). In a case of tissue samples, it is possible to detect characteristic *m*/*z* values, but additional peaks are also present.

### 2.2. Different Hexoses Analysis by Derivatisation with Betaine Aldehyde

**Figure 2:**
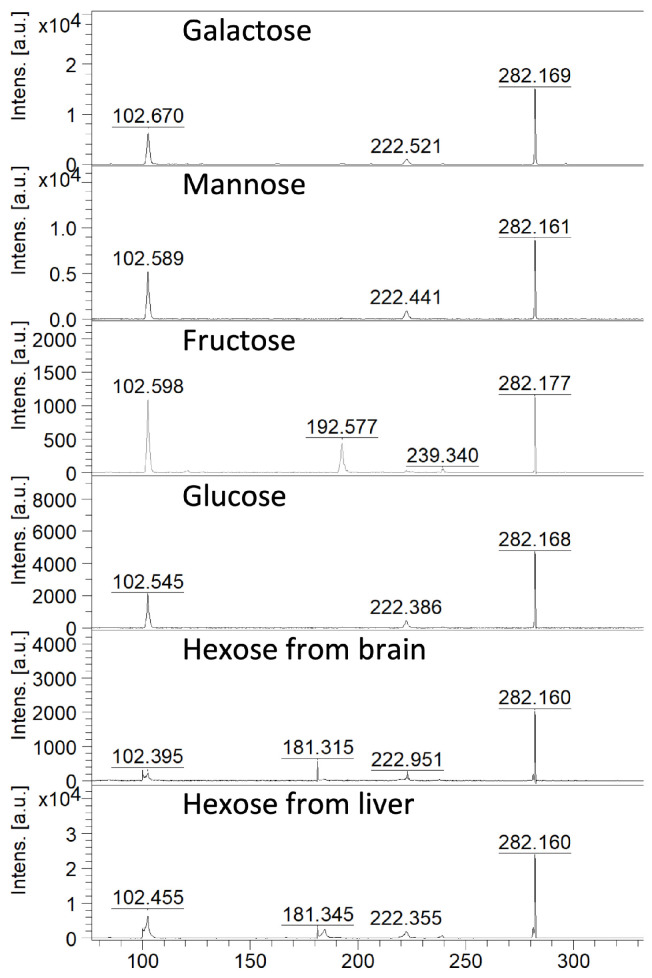
MALDI MS/MS spectra from galactose, mannose, fructose and glucose and additionally, from the brain and liver tissue after betaine aldehyde derivatisation.

Galactose, glucose, mannose, and fructose are all monosaccharide sugars that share the same chemical formula (C_6_H_12_O_6_), thus the same molecular mass, but differ in how their atoms are arranged. In the next experiment, we examined whether our method reacts differently with different hexoses. The results showed that only fructose yielded a different MS/MS spectrum. For galactose, glucose, and mannose, the resulting MS/MS spectra were the same (Figure 2). Since glucose is the most biologically important and predominant hexose in animal tissues (see Section 3—Discussion), we focused on it during quantitative analysis and calibration curve creation. However, it is important to remember that, when discussing a complex tissue, we cannot distinguish between different hexoses using our chemical derivatisation approach.

### 2.3. MSI Hexoses Analysis in the Brain and the Liver Tissue

**Figure 3:**
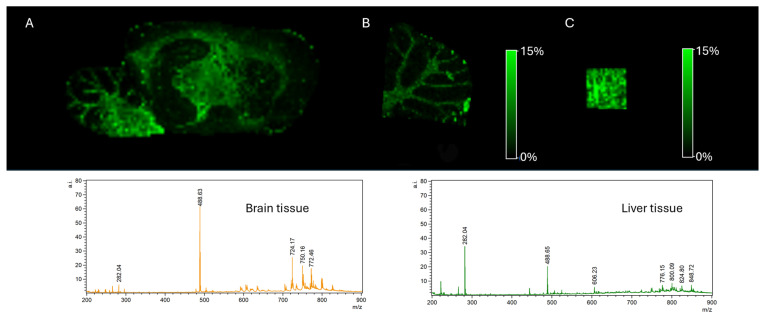
MALDI MSI of hexose (*m*/*z* = 282). (**A**) Sagittal section of a brain and (**B**) brain cerebellum, with appropriate spectrum, and (**C**) liver tissue with appropriate spectrum.

Studies to optimise the method were performed using liver and rat brain tissue sections. The betaine aldehyde-derivatised hexose signal varies by tissue. An MSI image of hexose distribution is shown in Figure 3, along with the corresponding spectra.

### 2.4. Analysing the Endogenous Substance with Its SIL Analogues—Calibration Curve

Since glucose, which prevails among hexoses, is an endogenous substance, we decided to use SIL analogues of glucose to obtain a proper calibration curve. In this case, we assume that the matrix effect will be similar for the endogenous substance and the labelled analogue. To prepare a biomimetic calibration curve for quantitative analysis, we prepared a homogenate spiked with D-glucose ^13^C_6_. To apply all calibration curve points at once (‘with one cut’), we used a boiled egg white as a mould for the homogenate with different isotope concentrations.

**Figure 4:**
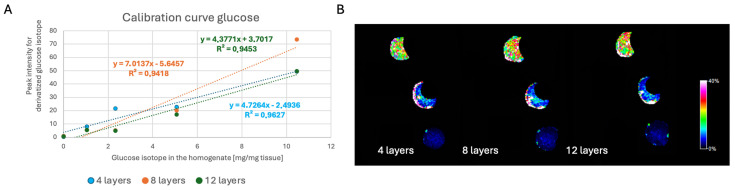
The influence of the number of betaine aldehyde layers (1 mg/mL) on the calibration curve. (**A**) Calibration curve for SIL D-glucose ^13^C_6_ with different numbers of betaine aldehyde layers and (**B**) tissue homogenate images for different numbers of betaine aldehyde layers.

To ensure that we used the correct amount of betaine aldehyde and that all the SIL D-glucose ^13^C_6_ from the tissue had reacted, we increased the number of betaine aldehyde layers. Thus, we tested 4, 8, and 12 layers of a 1 mg/mL betaine aldehyde solution. We obtained the best results with 8 layers of betaine aldehyde solution at a concentration of 1 mg/mL (see Figure 4). For 12 layers, the signal intensity was slightly lower. To reduce the risk of molecular delocalization, we decided to use two layers of betaine aldehyde solution at a concentration of 4 mg/mL.

### 2.5. The Influence of the Position of the Spraying Nozzle over the Tissue

To apply the betaine aldehyde solution and the CHCA matrix, we used the SunCollect system, which is recognised as a wet-interface system. It can spray the matrix solution from different heights over the sample. At the higher position (Z = 5 in the SunCollect system; Z is defined as the distance from the highest nozzle position), the driest vapor is produced, resulting in the finest matrix crystal size. At the lowest position (Z = 25 mm), a wetter, more atomised spray will achieve the target. This allows the matrix solution to penetrate the tissue, but it may also contribute to the delocalisation of molecules.

**Figure 5:**
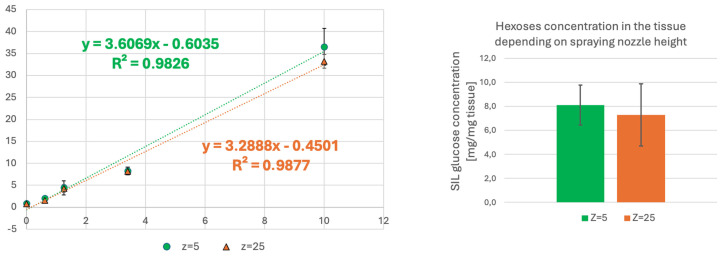
The influence of the position of the spraying nozzle over the tissue section.

Our analysis proved that the nozzle position during CHCA matrix application does not influence the results (see Figure 5). However, the standard deviation for the higher spraying nozzle position is lower.

### 2.6. Calibration Curve for Liver and Brain Analysis

**Figure 6:**
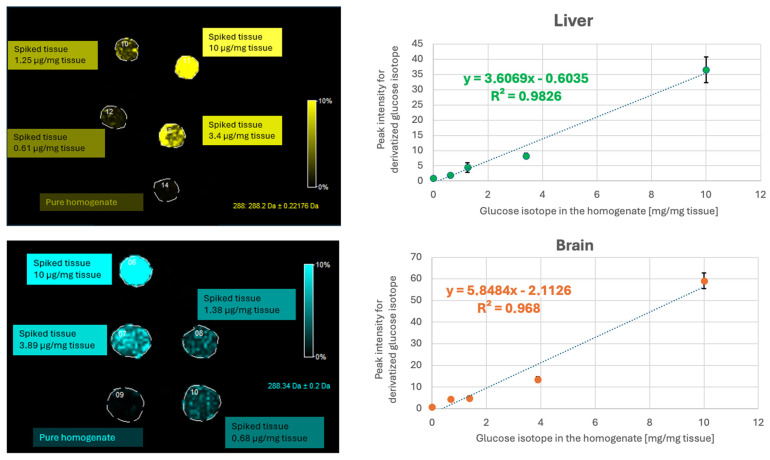
Calibration curve obtained using SIL D-glucose ^13^C_6_ derivatised with betaine aldehyde for liver (**upper** image) and brain (**lower** image). Samples were prepared using 2 layers of betaine aldehyde solution (4 mg/mL), followed by 10 layers of CHCA matrix solution (25 mg/mL). Both solutions were sprayed with the nozzle set to the higher position (Z = 5).

Ultimately, for the liver and the brain tissue, the most optimal analysis parameters were considered to be two layers of betaine aldehyde solution at a concentration of 4 mg/mL and the higher nozzle height (Z = 5) for derivatisation of SIL D-glucose ^13^C_6_ from the calibration curve and potential hexoses from the tissue (Figure 6).

### 2.7. Calibration Curve Prepared ‘Freshly’ and After 6-Day Storage

**Figure 7:**
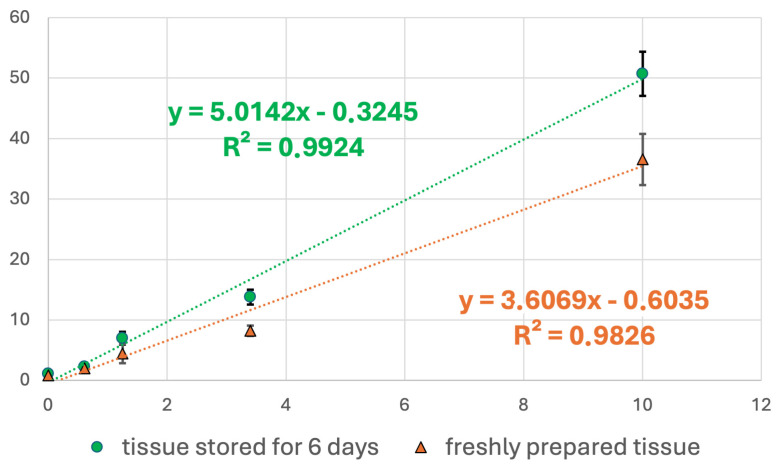
The problem of “calibration curve” storage.

Preparation of the calibration curve in that way is quite tedious. The most desirable option would be to prepare such material once and use it for subsequent measurements. Unfortunately, our research has shown that peak intensities for derivatised glucose at the calibration curve points differed when the prepared homogenates in the boiled egg white were stored for 6 days. Hence, care must be taken during such analysis (Figure 7). Apart from this section, all presented calibration curves and quantitative results relied on freshly prepared calibration samples.

### 2.8. Changes in Hexose Concentration Due to Morphine Administration

Morphine addiction is regulated by a complex network of brain regions and structures—some engaged in these processes are listed below. The ventral tegmental area (VTA) is a part of the mesolimbic dopamine system. It is responsible for the reinforcing effects of morphine since activation of dopaminergic neurons in this region is responsible for the initial rewarding effect of the drug. Nucleus accumbens (NAC) is a major target of VTA dopamine projections and plays an important role in mediating the rewarding properties of morphine. It is involved in the motivational aspects of drug-taking behaviour. The medial prefrontal cortex (mPFC) is responsible for regulating emotional and cognitive processes associated with addiction. The amygdala is engaged in drug-seeking behaviour. Their subregions are involved in the emotional and associative learning processes that link environmental cues with drug effects. The bed nucleus of the stria terminalis (BNST) is part of the extended amygdala and contributes to stress-related aspects of addiction. It may also play a role in relapses. The hippocampus is involved in the encoding and retrieval of contextual and spatial memory associated with drug use. It plays a key role in the acquisition of novel rewarding stimuli and in responding to previously learned drug-related cues. Although the thalamus is less well-characterised in the context of morphine addiction, it functions as a relay centre, integrating and transmitting sensory and limbic information to cortical and subcortical regions involved in addictive behaviours.

Due to limited access to animal tissues and the preliminary nature of this study, a sagittal tissue section from one addicted and one control animal, at 1.13 mm and 3.75 mm according to the Rat Brain Atlas, was performed to observe the changes in hexose levels in structures located at this plane. For the brains of the morphine-addicted and control rats, three regions of interest (ROIs) from the analysed structure were selected for comparison, and the average peak intensity and standard deviation were calculated.

**Figure 8:**
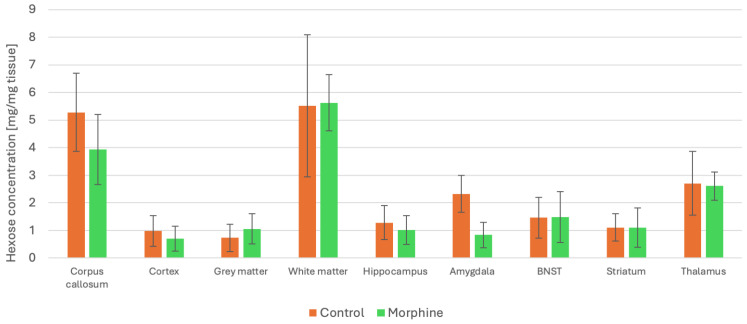
Comparison of hexose concentration in different brain structures after morphine administration.

Among all the structures, only the amygdala showed significant changes (see Figure 8). In the remaining structures, we observed some trends, but with substantial standard deviation. It should be noted that a single animal does not constitute biological replication and therefore cannot be used to infer statistical significance between groups. The brain data are presented as exploratory (pilot) observations.

Additionally, some measurements of hexose levels under morphine influence were performed in the peripheral tissues from the spinal cord, kidney, and liver. It was assumed that the spinal cord, as a part of the peripheral nervous system with a lot of mu receptors, could be vulnerable to changes influenced by morphine. Zaitsu et al. in their research have proved that cocaine, methamphetamine, and morphine may influence metabolites in urine, including tricarboxylic acid cycle intermediates, during morphine addiction. This may also suggest some disruption in kidney metabolism during drug addiction [10]. What is more, in their study, Payabvash et al. showed changes in liver glutathione concentrations, the glutathione synthesis pathway, and enzymatic antioxidant defence, revealing the pro-oxidative effects of chronic morphine treatment [11]. Since we had access to this type of tissue, we decided to check the hexose levels there as well. For the experiment, liver, one kidney, and spinal cord were taken from three different morphine-addicted and control animals.

**Figure 9:**
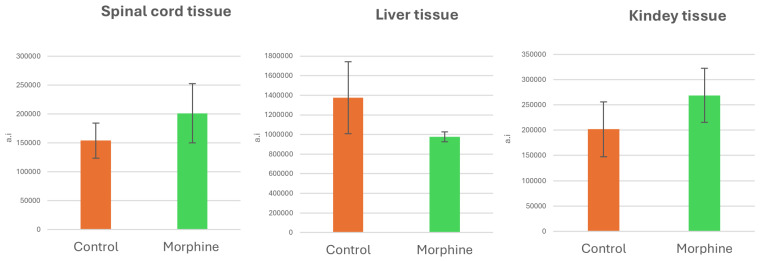
Comparison of MS peaks’ intensities for hexose in spinal cord, liver and kidney tissue after morphine exposition (n = 3).

Unfortunately, due to a relatively high standard deviation, we were unable to determine whether there is a significant trend in hexose levels after morphine administration (see Figure 9).

## 3. Discussion

Although MSI is recognised as a potent analytical technique that can identify and map ‘hundreds of molecules in a single experiment’ [12], we must remember that these molecules must be able to ionise.

Betaine aldehyde reacts selectively with the hydroxyl group. What is more important is that it exists as a quaternary-ammonium ion. Thus, it may be useful for facilitating the analysis of substances that cannot ionise in the MALDI ion source. Betaine aldehyde reacts with hexoses, forming a derivatisation product at *m*/*z* = 282.2. Unfortunately, it is impossible to specify which hexose this derivatisation agent reacts with, since they are isomers with identical formulas, but different structures. Although it may sound like a serious drawback of the method, we must consider the biochemical roles of different hexoses.

In the liver, skeletal muscles, and other target tissues, most galactose is metabolised through the Leloir pathway. Via this route, galactose can be activated to UDP-α-D-galactose (UDP-Gal) and be used as a precursor for glycosylation. Alternatively, it can be converted to UDPα-D-glucose (UDP-Glc), which then participates in glycogen synthesis or glycolysis, depending on the tissue type and its energy demand. Therefore, galactose plays a vital role as a structural component of glycoproteins and glycolipids. In its free form, galactose may be present only for a short time at low concentrations, as its accumulation may lead to increased production of galactitol, which acts as a metabotoxin, neurotoxin, and hepatotoxin [13]. The concentration of galactose in blood is about ten to a thousand times lower than that of glucose [14].

Diet and glycoprotein degradation may be the source of mannose. The plasma concentration of this hexose is about a thousand times lower than that of glucose [14,15]. Mannose is the primary monosaccharide component of N-glycans and is utilised for this purpose far more efficiently than glucose. It is also very quickly used for glycolysis after isomerisation to fructose-6-phosphate [16].

Therefore, glucose is the most abundant free sugar in animal tissues because it is responsible for energy metabolism, and it will likely be the primary substrate for betaine aldehyde derivatisation.

We have performed MSI analysis of brain and liver tissue sections using betaine aldehyde derivatisation and a CHCA matrix in positive ion mode. To perform quantitative analysis, we have used SIL D-glucose ^13^C_6_. Since both isotope and endogenous hexoses may be present in the sample, we optimised the amount of betaine aldehyde to ensure that all molecules react with the derivatisation agent. During the optimisation, we found that derivatisation requires the application of two layers of betaine aldehyde solution at a concentration of 4 mg/mL in ACN–H_2_O, 2:1 *v*/*v*, and then the standard CHCA matrix (5 mg/mL in ACN–H_2_O (1:1) and 0.2% of TFA) has to be applied. Additionally, we have tested different nozzle positions for CHCA matrix deposition and found that, in a wet-interface matrix deposition system, a higher nozzle position is preferable as it produces less variation in results.

Preparation of the calibration curve is quite tedious. Unfortunately, our analysis showed that the measurement results might differ over time during storage, even at −80 °C. By ‘whole sample’ we mean boiled egg white with homogenates spiked with SIL D-glucose ^13^C_6_. Hence, care must be taken during such analysis.

There are a few studies in the literature that address the problem of the influence of sample storage on MALDI MSI analysis over time. For example, Lukowski et al. investigated kidney tissue and the signals from different lipid species under various storage conditions. They found that signals from different lipid species decreased and that molecular degradation of the tissue sections was unavoidable over time, regardless of storage conditions. In storage conditions similar to ours (6 days, at −80 °C, glass slides stored in Ziploc bags, without any special protection), they reported a 40% drop in overlapping annotations in lipids between freshly prepared tissue and stored samples [17]. It is possible that artificially added and derivatised isotopes could affect the balance between ionised and non-ionised molecules, since the signal from the rest of the matrix is diminished. Additionally, it was shown that the freeze/thaw process may influence the intensity of different peaks in MS spectra [18]. In our case, the freshly prepared sample is frozen only to about −14 °C for cutting, whereas the ‘old’ sample is frozen after cutting to −80 °C, then stored, and warmed to −14 °C before the next sample preparation. This could also impact the observed signals.

Unfortunately, we do not have a satisfactory solution for this problem. Nevertheless, the observation is intriguing and definitely requires further examination.

Lastly, we checked whether we could observe the effect of morphine addiction in different peripheral tissues and whether we could measure hexose concentration in various structures of the brain. Considering peripheral tissues, we were unable to indicate any substantial differences. In the sagittal section of the brain, the amygdala was identified as the structure affected by morphine administration; however, the findings are preliminary and additional research is necessary in this area.

## 4. Materials and Methods

### 4.1. Materials

HPLC gradient-grade ACN, water, and MeOH were obtained from J.T. Baker (Amsterdam, The Netherlands); betaine aldehyde, trifluoric acid (TFA), and stable labelled isotope of glucose [^13^C_6_] were obtained from Sigma-Aldrich/Merck, Poland; The glucose was obtained from Agilent Technologies (Santa Clara, CA, USA).

### 4.2. Animal Treatment and Tissue Preparation

All experiments on animals were performed in accordance with the approval of the Polish and European Communities Council Directives (86/609/EEC) and were permitted by the Local Bioethics Committee for Animal Experiments at the University of Life Sciences in Lublin, approval code: 35/2020, date: 25 May 2020. In MSI experiments, only a portion of the animal tissue is typically used. The tissue is cut on a microtome at a specified location, and the residual material can be used in various ways. In our case, it was used to prepare a homogenate, which was then used to prepare a calibration curve. For method optimisation, tissues were obtained from control animals. Brains and livers were isolated and stored at −80 °C in a laboratory freezer (NU9483, NUAire Inc., Plymouth, MN, USA).

For experiments involving morphine administration, male Wistar rats, weighing approximately 250–300 g each (3 months old), were obtained from a local distributor (HZL, Warsaw, Poland). They were housed in groups of three under a 12/12-h light/dark cycle in an air-conditioned room. Water and standard laboratory food were freely available. Drug dependence was induced by subcutaneous (s.c) injection of morphine hydrochloride (Polfa, Poznan, Poland), at 10 mg/kg [19] with saline (0.9% NaCl) as a control, once a day for 10 days. After the last injection, the animals were sacrificed by decapitation. Brains were isolated from the skull and immediately frozen in liquid nitrogen. Then, tissues were stored in a Dewar (LS750, Wortington Industries, Columbus, OH, USA) until further processing. For the analysis, liver, one kidney, and spinal cord were taken from three different morphine-addicted and control animals. In the case of the brain from the morphine addicted and control rat, three different ROIs from the analysed structure were taken for comparison. The rats were randomly assigned to the control and morphine groups, but no specific randomisation method was used.

### 4.3. Calibration Curve

For quantitative analysis, we prepared a calibration curve based on liver and brain tissue homogenates. The final optimised maximum concentration of stable labelled isotope of glucose [^13^C_6_] (Sigma-Aldrich) was 10 ug per 1 mg of tissue. It was important not to dilute the tissue with added substances, so glucose was prepared as a concentrated solution in water (20 mg/mL), and only a minute volume of solution was added to the homogenate.

Initially, both tissues were thoroughly cut with the scalpel in different directions and homogenised in the glass homogeniser. The grinder consisted of a borosilicate glass cylinder (1 mL volume) and a conical PTFE (Polytetrafluoroethylene) pestle, with a pestle-cylinder clearance of 0.1 to 0.15 mm (Wheaton, Bionovo, Legnica, Poland), which was used for mechanical homogenization. After homogenization, all the tissue was removed from the glass cylinder with a short centrifugation ‘upside down’ in a 15 mL ‘Falcon’ tube (1600 rpm, a few seconds). A portion of the prepared tissue was transferred to a fresh probe of the Eppendorf type with the aid of a positive-displacement pipette (Pos-D) Ranin (Mettler Toledo), Warsaw, Poland and weighed. For the liver homogenate, the weight roughly corresponded to the homogenate volume, and for the final experiment, 67.27 mg of tissue was taken. For the brain, the ratio was more or less like 1:2, and for the final experiment, 81.82 mg of tissue was used. Glucose solution was added to the homogenate and mixed with a conical form PTFE pestle, and that was the point of maximum concentration in the calibration curve.

**Figure 10:**
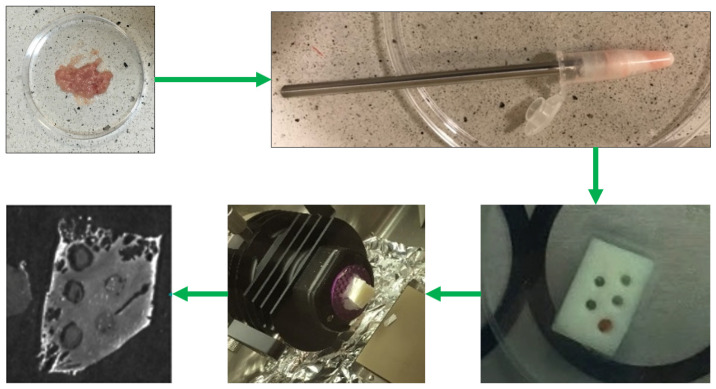
Sample preparation: from the spiked tissue homogenate, through calibration curve spots prepared in a mould made from boiled egg white, to the tissue slice on ITO glass.

The lower concentration points for the calibration curve were prepared as follows: in a clean Eppendorf tube a portion of the spiked homogenate was added to a portion of the clean homogenate (without isotope) tube with the aid of a positive-displacement pipette (Pos-D) in a ratio of approximately 1:1. The mass of each portion (the one with isotope and the ‘clean’ one) was thoroughly weighted to be able to precisely calculate the amount of D-glucose ^13^C_6_ in the ‘point’. Two portions of homogenates were mixed with the aid of a PTFE pestle to ensure even distribution of the isotope after their ‘dilution’ with the clean homogenate. Then, the tube was centrifuged to pull the tissue to the bottom of the Eppendorf tube and to prevent drying the material and losing the precious sample with the isotope. This procedure was repeated three times to obtain 0.5, 0.25, and 0.125 of the maximum glucose and isotope concentration. In this way, with the clean tissue (‘blank’), we obtained 5 points for our calibration curve. To apply all the calibration curve points at once (‘with one cut’), we used the white of a boiled egg with prepared holes as a type of mould for the homogenate mixtures. The boiled egg white was cut with a scalpel to obtain a cuboid. For the calibration curve, holes were made in the boiled egg white using a glass capillary (approximately 2 mm in diameter). The homogenate mixture was placed into the holes using a Pos-D pipette. The entire system was frozen and kept at −20 °C until sectioning (see Figure 10).

### 4.4. Tissue Sectioning

Generally, all the slices were prepared similarly. The different tissue sections were cut at 12 µm, placed on indium tin oxide (ITO) glass (Bruker Daltonics, Bremen, Germany) and immediately thaw-mounted. After cutting, the ITO glasses were vacuum dried for 15 min. After drying, the optical image (600 dpi) of the ITO glasses with dried tissues was recorded.

For the calibration curve, the boiled egg white was kept at −15 °C in a cryomicrotome chamber (Cryotome FSE, Thermo Fischer Scientific, Chesire, UK) for approximately 1 h before cutting to ensure the homogenate was frozen. Thin boiled egg white slices (12 µm) were placed on indium tin oxide (ITO) glasses (Bruker Daltonics, Bremen, Germany) and immediately thaw-mounted.

For the MS imaging experiments with rat brains under morphine influence, sagittal sections of the brain at 1.13 mm and 3.75 mm according to the Rat Brain Atlas [20] were taken. These coordinates allowed us to obtain brain sections through the main structures involved in morphine addiction (AMG, amygdala; BST, bed nucleus of the stria terminalis; cerebral cortex; corpus callosum; central lateral thalamic nucleus; hippocampus; thalamus).

### 4.5. Matrix Application

During experiments, a solution of betaine aldehyde and a CHCA matrix was applied using a SunCollect sprayer (SunChrom GmbH, Friedrichsdorf, Germany). The concentration of betaine aldehyde solution was 1 mg/mL or 4 mg/mL, ACN–H_2_O, 2:1 *v*/*v*. The flow rate of the betaine aldehyde solution was 60 µL/min. The nozzle sprayed betaine aldehyde at a line distance of 2 mm and a spray speed of 800 mm/min. Position of the spraying nozzle was Z = 5. Different layers (l) of betaine aldehyde were applied during sample preparation optimisation. After betaine application, the CHCA matrix was applied to the glass in 10 layers, and in some cases, the spraying nozzle was positioned at Z = 5 or Z = 25. The matrix solution was made at 5 mg/mL in ACN–H_2_O (1:1) containing 0.2% of TFA.

### 4.6. MALDI Measurement

Matrix-coated sections were subjected to imaging experiments using the MALDI–TOF/TOF UltrafleXtreme MS (Bruker-Daltonics, Bremen, Germany) with a Smartbeam II™ laser operating at 2 kHz. All following MS parameters underwent initial, multistep optimisation. Ions were accelerated at 25 kV with a pulsed ion extraction of 120 ns and ion suppression up to 100 Da. Spectra were recorded in positive and negative ion modes with a reflectron, within a 100–3000 *m*/*z* range. They were externally calibrated with Peptide Calibration Standard II (Bruker-Daltonics, Bremen, Germany) and known matrix ions. A raster width of 150 μm was applied to the brain samples, and a raster width of 100 μm was applied to the liver samples.

In total, 400 shots were collected from each ablation point, with 20 shots at the raster spot, and the laser focus diameter was set to ‘3_medium’. FlexControl version 3.4 (Bruker-Daltonics, Bremen, Germany) was used for spectra acquisition, and FlexImaging version 4.0 was used for data processing and molecular image creation. FlexAnalysis (version 3.4) was used for the spectra analysis. All the data were normalised to TIC.

### 4.7. MS/MS Analysis of Glucose from the Tissue

To confirm that we are measuring glucose from the tissue, we performed MS/MS analysis. We prepared samples of glucose (0.5 mg/mL) and glucose derivatised with betaine aldehyde in solution (0.5 mg/mL), and performed dried-droplet analysis with CHCA (5 mg/mL ACN–H_2_O, 2:1; 0.2% TFA). Analysis of liver, kidney, and spinal cord samples was performed using a Bruker RapifleX MALDI-ToF/ToF mass spectrometer (Bruker-Daltonics, Bremen, Germany) operated under the control of flexControl software (version 4.2). Reflector positive (RP) ion mode was employed within an acquisition range of 120–2000 *m*/*z*. The instrument was externally calibrated using the Peptide Calibration Standard II (Bruker Daltonics).

### 4.8. Hexoses Analysis by Derivatisation with Betaine Aldehyde

Dried droplet analysis was performed of 0.5 mg/mL of glucose, galactose, mannose, and fructose with betaine aldehyde derivatisation, and the results were compared with those from the tissue.

## Data Availability

The original contributions presented in this study are included in the article. Further inquiries can be directed to the corresponding author.

## Abbreviations

The following abbreviations are used in this manuscript:

ACN	Acetonitrile
CHCA	α-Cyano-4-hydroxycinnamic acid
DESI	Desorption Electrospray Ionization
ITO	Indium Tin Oxide glass
MALDI	Matrix laser desorption ionization
MSI	Mass Spectrometry Imaging
PTFE	Polytetrafluoroethylene
ROI	Region of interest
SIL	Stable isotope labelling
TFA	Trifluoroacetic Acid
